# Relationship between serum mineral levels in the second and third trimester of pregnancy and the risk of gestational diabetes mellitus: a retrospective cohort study

**DOI:** 10.3389/fnut.2025.1634419

**Published:** 2025-10-29

**Authors:** Yuxin Hao, Na Wang, Sumiao Hong, Yongyi Liu, Guankai Lin, Xiaoyang Xu, You Zhou, Xiaoting Wen, Baochang Sun, Hexing Wang, Min Huang, Jiwei Wang, Yue Chen, Qingwu Jiang

**Affiliations:** ^1^Key Lab of Health Technology Assessment of Ministry of Health, School of Public Health, Fudan University, Shanghai, China; ^2^Department of Obstetrics, The People's Hospital of Pingyang, Wenzhou, Zhejiang, China; ^3^Joseph L. Mailman School of Public Health, Columbia University, New York, NY, United States; ^4^Wenzhou Center for Disease Control and Prevention, Wenzhou, Zhejiang, China; ^5^School of Epidemiology and Public Health, Faculty of Medicine, University of Ottawa, Ottawa, ON, Canada

**Keywords:** mineral, gestational diabetes mellitus, micronutrients, gestational age, retrospective study

## Abstract

**Introduction:**

Gestational diabetes mellitus (GDM) poses significant health risks for both the mother and fetus, and it also increases the mother’s risk of developing type 2 diabetes later in life. Mineral elements may play a crucial role in the development of GDM by influencing insulin metabolism. However, comprehensive studies on serum mineral levels during pregnancy remain limited. This study aims to evaluate the relationship between serum mineral levels in pregnant women during the second and third trimesters and the risk of developing GDM.

**Methods:**

This retrospective cohort study included 17,224 singleton pregnancies delivered between 2016 and 2022 at a tertiary hospital in China. Maternal demographic data and serum mineral concentration information from the mid and late stages of pregnancy were collected through the hospital information system. Analyses were conducted using restricted cubic spline models and multivariate logistic regression models.

**Results:**

The prevalence of GDM in this study was 15.07%. Chloride [*P* for overall = 0.01; *P* for non-linear = 0.373; OR (95% CI) = 1.03 (1.01, 1.05)] showed a significant linear positive association with GDM. Additionally, serum levels of calcium (*P* for non-linear < 0.001), potassium (*P* for nonlinear = 0.036), and magnesium (P for nonlinear < 0.001) were found to have non-linear relationships with the risk of GDM. The interactions between calcium and magnesium [OR (95% CI) = 0.05 (0.01, 0.27), *P* for interaction < 0.001], potassium and magnesium [OR (95% CI) = 0.11 (0.03, 0.37), *P* for interaction < 0.001], and potassium and chloride [OR (95% CI) = 1.06 (1.01, 1.11), *P* for interaction < 0.001] were significant.

**Discussion:**

The study indicates that specific serum mineral levels in pregnant women are closely associated with the risk of gestational diabetes mellitus. A deeper understanding of the mechanisms and interactions of these minerals could aid in developing effective prevention and treatment strategies, thereby reducing the incidence of GDM and improving pregnancy outcomes.

## 1 Introduction

Gestational diabetes mellitus (GDM) is a type of diabetes that first appears or is diagnosed during pregnancy due to impaired glucose tolerance and is one of the most common metabolic disorders in pregnant women (1). GDM can lead to severe adverse health outcomes for both the mother and fetus, including macrosomia, low birth weight, and congenital abnormalities (2). Additionally, women with GDM have a higher risk of developing type 2 diabetes (T2DM) later in life (3). In 2021, the International Diabetes Federation (IDF) released a report titled “Global Diabetes Map,” which indicated that the global prevalence of GDM is approximately 16.7% (4). In China, the overall prevalence of GDM is about 14.8% (5). However, with the recent implementation of the two-child and even three-child policies, the increasing number of elder pregnant women, and improvements in economic development, living standards, and lifestyle changes, the number of GDM patients in China continues to rise (6).

In addition to traditional risk factors such as overweight and obesity, advanced maternal age, ethnicity, history of GDM, and a family history of T2DM, mineral elements may also play a significant role in the occurrence of GDM (7–9). Minerals may influence the occurrence of GDM through their effects on the secretion, synthesis, storage, and metabolism of insulin (10). Trace elements are closely related to the incidence of GDM. Insufficient intake of trace elements can impair cellular and tissue functions, leading to GDM and pregnancy-related hypertension (11). Some studies have shown that the plasma level of zinc (Zn) is negatively correlated with GDM risk, with Zn promoting the development of GDM by disrupting insulin metabolism and glucose homeostasis (12, 13). Other research suggests that high levels of iron (Fe) and copper (Cu) increase the incidence of GDM (14). Elevated Fe levels can induce oxidative stress, β-cell toxicity, and insulin resistance, potentially leading to an increased risk of GDM (15).

It is noteworthy that macroelements also play an important role in glucose metabolism and insulin function. Evidence indicates that deficiencies in calcium and magnesium may increase the risk of GDM by impairing insulin secretion and promoting insulin resistance (16, 17). In addition, elevated potassium levels and imbalances between sodium and potassium have been associated with a higher risk of GDM (18, 19). Abnormal phosphate levels and disturbances in chloride balance may also indirectly influence insulin action by affecting energy metabolism and acid–base homeostasis (20, 21). Therefore, specific abnormalities in both trace and macroelements levels may contribute to the development of GDM through their effects on insulin function and glucose metabolism.

Although the impact of trace elements on GDM has been extensively studied, there is a lack of research on the relationship and interactions between macroelements in minerals and GDM. Therefore, investigating this relationship is of considerable significance. Hence, we conducted a large-scale retrospective epidemiological study to examine the association between serum mineral levels in the second and third trimesters of pregnancy and the occurrence of GDM.

## 2 Materials and methods

### 2.1 Study population

This study was a retrospective cohort study involving 17,224 pregnant women at the People’s Hospital of Pingyang, Wenzhou City, Zhejiang Province, China, conducted between January 2016 and December 2022. The hospital is the primary provider of medical services in Pingyang County and operates as the only tertiary hospital in the area. Most pregnant women in the region choose this hospital for delivery. The hospital has an effective information system that archives demographic and clinical data for each patient. In this study, all participants underwent pregnancy registration and examination, with all cases involving singleton deliveries.

Inclusion Criteria: (1) Met the diagnostic criteria for GDM (22); (2) Singleton pregnancies; (3) Natural conception or *in vitro* fertilization; (4) Received routine prenatal care.

Exclusion Criteria: (1) Diabetes complicating pregnancy (pre-existing diabetes); (2) Pregnancy-related hypertensive disorders, including chronic hypertension (pre-existing), gestational hypertension, preeclampsia, and eclampsia; (3) Missing data and other reasons.

The study was approved by the Medical Research Ethics Committee of the School of Public Health, Fudan University (The international registry no. IRB00002408 and FWA00002399). This retrospective study utilized existing data with all personally identifiable information removed. Informed consent was not required, which was also approved by the Medical Research Ethics Committee of the School of Public Health, Fudan University.

### 2.2 Data collection and measurement

The demographic and clinical data were obtained from the hospital information system.

The diagnostic criteria for GDM, as recommended by the Diabetes and Pregnancy Study Group (IADPSG), are based on the oral glucose tolerance test (OGTT) conducted between 24 and 28 weeks of gestation. GDM was diagnosed if any of the following criteria were met: fasting plasma glucose ≥ 5.1 mmol/L (≥92 mg/dL), 1-h plasma glucose ≥ 10.0 mmol/L (≥180 mg/dL), or 2-h plasma glucose ≥ 8.5 mmol/L (≥153 mg/dL) (23).

Mineral information was obtained from the prenatal check-up records before hospitalization for delivery, covering both the mid and late stages of pregnancy. Blood samples were collected from the antecubital vein of fasting pregnant women (fasting for more than 8 h) using 5 mL BD vacutainer tubes (Becton Dickinson, Franklin Lakes, NJ, United States). After allowing the blood samples to clot, they were centrifuged at 3,500 rpm for 10 min. The serum samples were then processed to remove any interference from hemolysis, jaundice, and lipid turbidity. Serum sodium (Na), potassium (K), chloride (Cl), calcium (Ca), magnesium (Mg), and phosphate (P) were measured on the ARCHITECT c16000 clinical chemistry analyzer (Abbott Diagnostics, Abbott Park, United States). Na, K, and Cl levels were measured using ion-selective electrode methods; Ca levels were determined using the Arsenazo III method; Mg levels were assessed using enzymatic methods; and P were measured using the molybdenum blue method; According to published literature and Abbott manufacturer data, using three levels of commercial quality control sera (low, medium, and high), within-run CVs for these analytes ranged from 0.5–1.5% and between-run CVs from 1.0–2.0% (24, 25), indicating that the system exhibits good analytical precision across different concentration levels.

### 2.3 Covariates

Covariates included age, parity (previous live births), *in vitro* fertilization (IVF), and neonatal sex. Information on age and parity was recorded by the physician during the first pregnancy registration visit.

### 2.4 Statistical analysis

**Figure 1:**
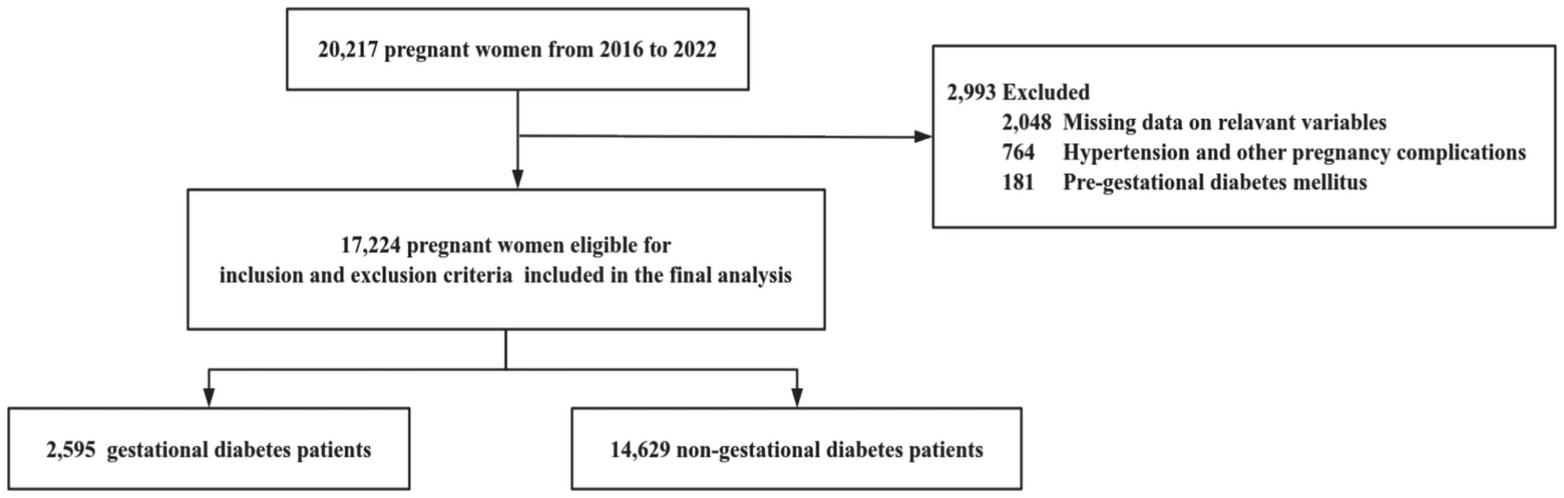
Flowchart of study participants.

Statistical analyses were conducted using R 4.2.3^1^. Continuous variables were reported as means with standard deviations (SD) or medians with interquartile ranges (IQR). Categorical variables were presented as frequencies and percentages. We investigated the dose–response relationship between maternal mineral levels and GDM risk using restricted cubic splines with four knots. The number of knots used in the restricted cubic spline analysis was selected based on the minimum Akaike Information Criterion (AIC). Restricted cubic splines were chosen because they can flexibly capture nonlinear relationships while maintaining linearity beyond the data boundaries, thereby avoiding unreasonable extrapolation and overfitting. This approach allows for more accurate and interpretable models, better reflecting the complex relationship between maternal mineral levels and GDM risk. Given the non-direct interpretability of the regression parameters from the spline regression (26), we used binary logistic regression models to derive interpretable parameters for linear relationships. For nonlinear relationships, we constructed piecewise logistic regression models to estimate inflection points and analyze threshold effects. In logistic regression models, we computed odds ratios (ORs) and 95% confidence intervals (CIs) associated with a one standard deviation change in mineral levels. Restricted cubic spline analysis and logistic regression analysis adjusted for age, parity (previous live births), IVF, and neonatal sex. A *p*-value less than 0.05 was considered statistically significant (Figure 1).

## 3 Results

### 3.1 Characteristics of study participants

This study included a total of 17,224 singleton mothers, with an average delivery age of 30.0 ± 4.79 years, of whom 16.26% were of advanced maternal age (≥35 years). Among the participants, 38.25% were primiparous, and 1.78% had undergone *in vitro* fertilization (IVF). The average serum levels of minerals during mid or late pregnancy were as follows: Ca 2.20 ± 0.16 mmol/L, Na 137.4 ± 1.84 mmol/L, K 3.86 ± 0.28 mmol/L, Mg 0.79 ± 0.12 mmol/L, P 1.24 ± 0.21 mmol/L, and Cl 103.7 ± 2.54 mmol/L. The median gestational age at delivery was 39.0 weeks (range: 38.0–40.0 weeks), and 15.07% of the participants were diagnosed with GDM (see Table 1).

**Table 1:** Characteristics of participants.

Characteristics	All participants
Participants, *n*	17,224
Maternal age, mean (SD), y	30.0(4.79)
Maternal age group, *n* (%), y	
<35	14,423 (83.74%)
≧35	2,801 (16.26%)
*In vitro* fertilization, *n* (%)	306 (1.78%)
Calcium, mean (SD), mmol/L	2.20 (0.16)
Sodium, mean (SD), mmol/L	137.4(1.84)
Potassium, mean (SD), mmol/L	3.86 (0.28)
Magnesium, mean (SD), mmol/L	0.79 (0.12)
Phosphorus, mean (SD), mmol/L	1.24 (0.21)
Chlorine, mean (SD), mmol/L	103.7 (2.54)
Neonatal sex, *n* (%)	
Male	9,360 (54.34%)
Female	7,864 (45.66%)
Parity group, *n* (%)	
None	6,588 (38.25%)
1–2	10,543 (61.21%)
3 or more	93 (0.54%)
Gestational age at delivery, median (IQR), wk	39.0 (38.0–40.0)
Gestational diabetes mellitus, *n* (%)	2,595 (15.07%)

SD, standard deviation; IQR, interquartile range.

### 3.2 Relationship between mineral levels and GDM

**Figure 2:**
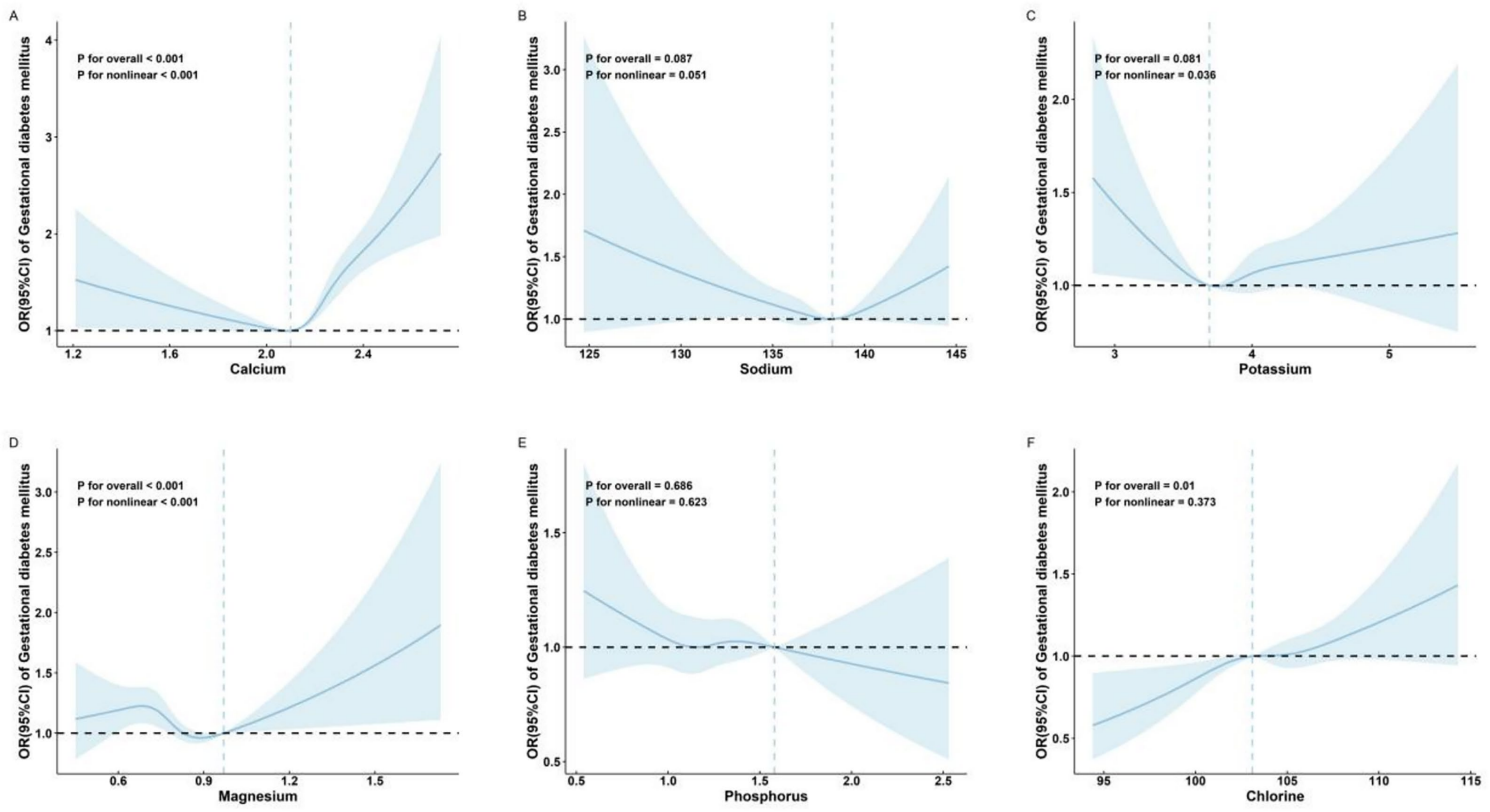
Adjusted* odds ratios and 95% confidence intervals for GDM in association with maternal serum mineral levels in restricted cubic spline regression. **(A)** calcium; **(B)** sodium; **(C)** potassium; **(D)** magnesium; **(E)** phosphorus; and **(F)** chlorine. The *x*-axis represents the full range of measured serum mineral levels to comprehensively display the data distribution captured by the restricted cubic spline model. *Adjusted for age (continuous), parity, *in vitro* fertilization, and neonatal sex.

Restricted cubic spline regression analysis showed nonlinear relationships between serum levels of Ca (*P* for non-linear < 0.001; Figure 2A) and GDM risk. The inflection point for Ca was 2.10 mmol/L. Ca levels below the inflection point were associated with a protective effect against GDM (OR: 0.61; 95% CI: 0.37, 0.99) (Table 2), while levels above the inflection point were associated with an increased risk of GDM (OR: 7.19; 95% CI: 4.49, 11.5) (Table 2).

**Table 2:** Adjusted* odds ratios (ORs) and 95% confidence intervals (95% CIs) for GDM in association with maternal serum mineral levels in multivariable logistic regression.

Serum mineral level	Inflection point (mmol/L)	Group (mmol/L)	OR (95%CI)	*p*-value
Ca^a^	2.10	≤2.10	0.61(0.37,0.99)	0.047
		>2.10	7.19(4.49,11.5)	<0.001
Na	–	–	NA	0.087
K^a^	3.69	≤3.69	0.56(0.32,0.99)	0.045
		>3.69	1.24(0.98,1.57)	0.068
Mg^a^	0.97	≤0.97	0.29(0.18,0.47)	<0.001
		>0.97	3.90(1.32,11.5)	0.014
P	–	–	NA	0.686
Cl	–	–	1.03(1.01,1.05)	0.002

“–” indicates no inflection point detected. “NA” indicates no significant association (*p* > 0.05).

ᵃPiecewise logistic regression.

*Adjusted for age (continuous), parity, *in vitro* fertilization, and neonatal sex.

No significant association was observed between Na (*P* for overall = 0.087; Figure 2B) and GDM risk.

Restricted cubic spline regression analysis also showed a nonlinear relationship between serum levels of K (*P* for non-linear = 0.036; Figure 2C) and Mg (*P* for non-linear < 0.001; Figure 2D) and GDM risk.

No significant association was observed between P (*P* for overall = 0.686; Figure 2E) and GDM risk.

The Cl was significantly linearly positively associated with GDM risk [*P* for overall = 0.01; *P* for non-linear = 0.373; OR (95% CI) = 1.03 (1.01, 1.05); Table 2 and Figure 2F].

### 3.3 Interaction between maternal serum mineral levels and risk of GDM

To further explore the associations between maternal mineral levels and GDM, we focused on minerals (Ca, K, Mg, and Cl) that demonstrated significant independent associations with GDM in our primary analyses and incorporated their interaction terms as independent variables in multivariable logistic regression models. After controlling for confounding factors such as age (continuous), parity, *in vitro* fertilization, and newborn sex, we found significant interactions between maternal serum Ca and Mg (OR: 0.05; 95% CI: 0.01, 0.27; *P* for interaction < 0.001) (Table 3). This indicates that when both Ca and Mg levels are elevated, their combined effect on GDM is negative, meaning that the joint effect of Ca and Mg significantly reduces the risk of GDM after adjusting for other variables. Similarly, the interaction between K and Mg was also significant (OR: 0.11; 95% CI: 0.03, 0.37; *P* for interaction < 0.001) (Table 3), suggesting that the combined effect of K and Mg significantly lowers the risk of GDM after controlling for other variables. The interaction between K and Cl was significant (OR: 1.06; 95% CI: 1.01, 1.11; *P* for interaction < 0.001) (Table 3), but the combined effect of K and Cl significantly increased the risk of GDM.

**Table 3:** Adjusted* the relationship between the risk of GDM and the multiplicative interaction of maternal serum mineral levels with odds ratio (ORs) and 95% confidence interval (95%CI).

Variable	OR (95%CI)	*P*-value
Ca × K	2.23(0.80,6.20)	0.126
Ca × Mg	0.05(0.01,0.27)	<0.001
Ca × Cl	0.96(0.86,1.07)	0.443
K × Mg	0.11(0.03,0.37)	<0.001
K × Cl	1.06(1.01,1.11)	0.023
Mg × Cl	1.09(0.95,1.26)	0.231

*Adjusted for age (continuous), parity, *in vitro* fertilization, and neonatal sex.

## 4 Discussion

We conducted a large-scale retrospective epidemiological study involving 17,224 singleton pregnancies. After adjusting for various confounding variables, our study revealed significant associations between maternal serum levels of Ca, K, Mg, and Cl with GDM. Notably, interactions between Ca and Mg, as well as K and Mg, were negatively associated with GDM risk, while the interaction between K and Cl was positively associated with GDM risk.

Our findings indicated that elevated maternal serum Cl levels are associated with an increased risk of GDM. Cl, primarily present in the body as chloride ions, plays essential roles in maintaining fluid balance, contributing to gastric acid formation, and helping to regulate acid–base balance. While some studies have reported increased Cl levels due to exposure to organic pollutants in pregnant women (27, 28), direct research on the relationship between Cl and GDM is limited. Electrolyte imbalances can lead to metabolic disturbances, potentially impacting insulin sensitivity and secretion (29). However, Cl’s role in overall electrolyte balance suggests it may indirectly affect insulin function and glucose regulation.

Ca, the most abundant mineral in the body, is crucial for insulin secretion and intracellular signaling, helping to stabilize blood glucose levels (30). Our study found a U-shaped relationship between maternal serum Ca levels and GDM risk. Specifically, when maternal serum Ca levels fall below a certain threshold, the risk of GDM increases. During the second and third trimesters of pregnancy, Ca absorption in pregnant women significantly increases, particularly when dietary intake is low (31). Ca metabolism is influenced by both active (1,25(OH)₂D) and inactive (25(OH)D) forms of vitamin D. During pregnancy, although serum 25(OH)D levels remain unchanged, increased conversion to 1,25(OH)₂D due to elevated 1-α-hydroxylase activity in the placenta doubles serum 1,25(OH)₂D levels, enhancing Ca absorption (32, 33). Consequently, Ca deficiency could disrupt these processes, increasing insulin resistance and GDM risk. Conversely, elevated Ca levels above a critical threshold may also increase GDM risk, potentially due to hypercalcemia, kidney stones, alkalosis, and renal failure (34). While there is no strong evidence or large-scale clinical studies supporting the link between excessive Ca intake and increased GDM risk, some studies suggest that Ca supplementation may impact metabolic health, including insulin sensitivity and glucose metabolism (35), and could potentially affect the risk of GDM under certain conditions. The risk of GDM is lowest within a moderate range of serum Ca levels. A randomized, placebo-controlled study found that Ca plus vitamin D supplementation had beneficial effects on the metabolic status of women with GDM, which indicated that maintaining Ca levels within an optimal range is crucial for lowering GDM risk (36, 37).

Mg is an essential mineral involved in regulating body temperature, synthesizing proteins and nucleic acids, and maintaining cellular electrical potentials (38). Our study showed that, up to a Mg level of 0.97 mmol/L, an increase in Mg levels is associated with a transient rise in the risk of GDM followed by a decrease. However, beyond this threshold, higher Mg levels are linked to an increased risk of GDM. Mg participates in numerous enzymatic reactions, including those related to glucose metabolism and insulin secretion, which is vital for maintaining normal blood glucose levels and insulin function (39). Low Mg levels have been associated with insulin resistance and metabolic syndrome, as Mg regulates ion channels, cellular processes, and physiological functions and is involved in insulin signaling pathways (40). Reduced intracellular Mg^2+^ can impair insulin receptor (IR) activity, damage post-receptor effects, and increase insulin resistance, all of which are risk factors for GDM (17). Mg supplementation may help improve insulin sensitivity and reduce GDM risk. Recent meta-analyses have shown that Mg supplementation positively affects glucose homeostasis in GDM patients and may reduce preeclampsia and increase neonatal birth weight (41–43).

K helps maintain fluid balance inside and outside cells and is involved in insulin secretion and function. It also plays a crucial role in regulating blood pressure and glucose levels (44). Our study found that low K levels are protective against GDM. Low K levels at the start of pregnancy are associated with a significantly reduced risk of GDM. Early pregnancy K levels can predict the risk of severe pregnancy complications such as GDM and preeclampsia (18). However, some studies suggest that low K levels in non-pregnant individuals are linked to an increased risk of type 2 diabetes and hypertension (45, 46). Ensuring adequate K intake may help mitigate these risks, but further research is needed to confirm the role of serum K levels as predictors of GDM.

Our study found that maternal serum levels of Ca, Mg, K, and Cl significantly affect GDM risk both independently and through their interactions. A randomized, double-blind, placebo-controlled trial indicated that Mg-Zn-Ca-vitamin D supplementation has beneficial effects on glucose control in GDM patients (47). It has been suggested that Ca and Mg, or K and Mg, may enhance insulin sensitivity, regulate IR and glucose transporter (GLUT4) activity, and exhibit anti-inflammatory and antioxidant effects (42, 48). These combined effects may reduce insulin resistance caused by inflammation and oxidative stress, protect pancreatic β-cell function, and stabilize blood glucose levels, thereby lowering GDM risk. Additionally, Mg facilitates the absorption and utilization of K within cells, helping to maintain electrolyte balance and the stability of cellular functions (49). In contrast, the interaction between K and chloride positively correlates with GDM risk, possibly due to their combined impact on intracellular electrolyte balance. Excessive chloride may interfere with K function, leading to IR dysfunction and impaired insulin signaling, thus increasing insulin resistance (50). Chloride may also promote inflammatory responses, exacerbating insulin resistance (51). Traditionally, healthcare providers have primarily focused on the supplementation and monitoring of trace elements like folic acid, iron, and vitamins during pregnancy. However, our results suggest that the levels of these major minerals should also be given equal attention. Additionally, rather than focusing solely on individual mineral levels, it is important to consider the interactions between different minerals and their impact on GDM risk.

## 5 Limitations

There are several limitations of our study. First, as a retrospective cohort study, we were unable to collect data on key confounding factors such as pre-pregnancy body mass index (BMI), polycystic ovary syndrome (PCOS), and socioeconomic status. The lack of detailed information on vitamin and nutritional supplement use, including specific dosages of calcium, iron, and vitamin D, also limited our ability to fully assess their impact on GDM development. Second, our analysis was based solely on serum mineral concentrations, which may not fully reflect the true mineral status throughout the body, as these elements are differentially distributed across organs and tissues. Finally, our findings are subject to selection bias because we excluded pregnant women with complications who were likely to have had additional mineral tests. Given that these high-risk individuals often have more pronounced electrolyte or mineral abnormalities and are more prone to GDM, their exclusion may have underestimated the true association between mineral levels and GDM risk (52–54). Consequently, the generalizability of our results is limited to a general population of pregnant women without severe complications. Additionally, as mineral levels were measured only in a small proportion of individuals during early pregnancy, our analysis was largely confined to data from the second and third trimesters. Future studies should therefore explore the dynamic relationship between mineral status and GDM risk across different stages of gestation.

## 6 Conclusion

Our study found significant associations between certain maternal mineral levels and the risk of GDM. Specifically, the balance of minerals such as Ca, K, Mg, and Cl plays a crucial role in the prevention and management of GDM. Therefore, healthcare providers should closely monitor these mineral levels during pregnancy to ensure that expectant mothers receive adequate and balanced intake of minerals to reduce the risk of GDM. Furthermore, larger and higher-quality studies are needed to confirm these associations and explore the underlying mechanisms.

## Data Availability

The original contributions presented in the study are included in the article/supplementary material, further inquiries can be directed to the corresponding authors.

## Footnotes

^1^ http://www.R-project.org
